# Lentivirus-mediated expression of human secreted amyloid precursor protein-alpha prevents development of memory and plasticity deficits in a mouse model of Alzheimer's disease

**DOI:** 10.1186/s13041-018-0348-9

**Published:** 2018-02-09

**Authors:** Valerie T. Y. Tan, Bruce G. Mockett, Shane M. Ohline, Karen D. Parfitt, Hollie E. Wicky, Katie Peppercorn, Lucia Schoderboeck, Mohamad Fairuz bin Yahaya, Warren P. Tate, Stephanie M. Hughes, Wickliffe C. Abraham

**Affiliations:** 1 Department of Psychology, University of Otago, Box 56, Dunedin, 9054 New Zealand; 2 Department of Biochemistry, Brain Health Research Centre, Brain Research New Zealand, University of Otago, Box 56, Dunedin, 9054 New Zealand; 3 Department of Neuroscience, Pomona College, Claremont, California 91711 USA

**Keywords:** Amyloid precursor protein, Lentivirus, Hippocampus, Memory, Long-term potentiation, Amyloid, APP/PS1 mouse

## Abstract

Alzheimer’s disease (AD) is a neurodegenerative disease driven in large part by accumulated deposits in the brain of the amyloid precursor protein (APP) cleavage product amyloid-β peptide (Aβ). However, AD is also characterised by reductions in secreted amyloid precursor protein-alpha (sAPPα), an alternative cleavage product of APP. In contrast to the neurotoxicity of accumulated Αβ, sAPPα has many neuroprotective and neurotrophic properties. Increasing sAPPα levels has the potential to serve as a therapeutic treatment that mitigates the effects of Aβ and rescue cognitive function. Here we tested the hypothesis that lentivirus-mediated expression of a human sAPPα construct in a mouse model of AD (APPswe/PS1dE9), begun before the onset of plaque pathology, could prevent later behavioural and electrophysiological deficits. Male mice were given bilateral intra-hippocampal injections at 4 months of age and tested 8–10 months later. Transgenic mice expressing sAPPα performed significantly better than untreated littermates in all aspects of the spatial water maze task. Expression of sAPPα also resulted in partial rescue of long-term potentiation (LTP), tested in vitro. These improvements occurred in the absence of changes in amyloid pathology. Supporting these findings on LTP, lentiviral-mediated expression of sAPPα for 3 months from 10 months of age, or acute sAPPα treatment in hippocampal slices from 18 to 20 months old transgenic mice, completely reversed the deficits in LTP. Together these findings suggest that sAPPα has wide potential to act as either a preventative or restorative therapeutic treatment in AD by mitigating the effects of Aβ toxicity and enhancing cognitive reserve.

## Introduction

Alzheimer’s disease (AD) is a neurodegenerative condition resulting in part from increased β-secretase cleavage of amyloid precursor protein (APP) and a concomitant increase in amyloid-β (Aβ) over the alternative α-secretase cleavage products [1, 2]. A key α-secretase cleavage product is the neuroprotective protein termed secreted amyloid precursor protein-alpha (sAPPα). Since the α-secretase cleavage site lies within the Aβ sequence, production of Aβ and sAPPα are mutually exclusive from the same molecule of APP. In AD, the shift towards β-secretase cleavage and Aβ accumulation appears to be associated with reduced sAPPα production in the brain [3, 4].

The reduction in sAPPα levels could serve as a significant compounding factor in the disease process as sAPPα exerts beneficial physiological, biochemical and behavioural effects that may mitigate the detrimental effects of Aβ accumulation. These effects include neuroprotection [5, 6], enhanced neuronal development [7, 8], facilitated long-term potentiation (LTP; [9–11], enhanced protein synthesis [12], enhanced memory [13] and rescue of spatial memory deficits induced by α-secretase inhibition [10] or APP knockout [11]. In contrast, reduced cerebrospinal fluid sAPPα levels correlate with poor memory performance in both aged humans and rats [14, 15]. The consistent role that sAPPα plays in neuroprotection and memory formation raises the possibility that elevating sAPPα levels in the damaged or diseased brain may be a useful therapeutic approach [16–19].

The progressive nature of AD presents the opportunity to test the ability of therapies to prevent the initial onset and progression of cognitive impairments, versus reversing or ameliorating cognitive impairments associated with moderate to advanced AD. Using the latter approach, expression of rodent sAPPα via AAV9 in the hippocampus of 12–13 month old APPswe/PS1dE9 mice largely reversed observed impairments in LTP, partially reduced plaque load and rescued spatial reference memory [20]. This result provides hope for a sAPPα-based therapy commencing even after frank disease onset. Should predictive biomarkers become available, however, it would potentially be possible to deliver therapeutic solutions earlier, before any cognitive decline begins, and thus be of even more value. However, it is not yet known whether raising sAPPα concentrations prior to disease pathology becoming evident can prevent or ameliorate AD-like symptoms.

In the present study, we used a lentiviral vector to express human sAPPα in the hippocampus of young adult APPswe/PS1dE9 mice to evaluate the potential of sAPPα to prevent the age-related onset of AD-associated neuropathologies and cognitive deficits. We found that sAPPα expression prevented deficits in spatial reference and working memory, as well as a partial rescue of the LTP deficit, even in the absence of an effect on Aβ accumulation and plaque load. Chronic expression commencing later in life, or acute delivery of sAPPα in aged transgenic mice, also rescued LTP. These findings demonstrate that elevating sAPPα levels in the presymptomatic phase has therapeutic potential for AD, and extends previous data [20] that either chronic or acute delivery of sAPPα delivery after symptom development may also be efficacious.

## Methods

### Animals

Male B6C3-Tg (APPswe, PSEN1dE9)85Dbo/Mmjax transgenic hemizygous and wild-type (WT) littermates (The Jackson Laboratory, Bar Harbor, USA, https://www.jax.org/strain/004462) were maintained as a colony at the University of Otago. Animals were group-housed in standard caging until surgery at either 4 or 10 months of age. They were transferred to single housing at ~ 8 months of age to prevent injury from fighting between the males. Food and water were available ad libitum, and the cage contained one red plastic tube (approximately 5 cm in diameter, 10 cm long) and shredded paper bedding as standard housing. Animals were kept on a 12 h light:dark cycle (lights on at 7 am), and the room temperature was controlled via a thermostat set at 21 °C. All procedures were approved by the University of Otago Animal Ethics Committee and conducted in accordance with New Zealand Animal Welfare and Biosecurity Legislation.

Genotyping was carried out on tail tips which were lysed overnight at 55 °C in lysis buffer (100 mM Tris HCl pH 8.5, 5 mM EDTA, 0.2% (*w*/*v*) SDS, 20 mM NaCl) containing 20 μg/ml proteinase K. Isopropanol extracted DNA pellets were dissolved in TE buffer pH 8.0 (10 mM Tris, 1 mM EDTA). Polymerase chain reactions using two sets of primers that amplify the Psen transgene and mouse DNA as a positive control were carried out to distinguish between wild-type and transgenic animals. Primer sequences were obtained from the Jackson Laboratory (PsenTg_forward oIMR1644 AAT AGA GAA CGG CAG GAG CA, PsenTg_reverse oIMR1645 GCC ATG AGG GCA CTA ATC AT, control_forward oIMR7338 CTA GGC CAC AGA ATT GAA AGA TCT, control_reverse oIMR739 GTA GGT GGA AAT TCT AGC ATC ATC C). Agarose gel electrophoresis stained with ethidium bromide showed either one band that indicated a wildtype animal or two bands indicating a transgenic animal.

### Lentivirus (LV)

Approval for the packaging and use of recombinant lentiviral vectors was obtained from the Environmental Protection Agency, NZ (GMD03091). The HIV-1 derived lentiviral plasmid, pCDH-EF1-MCS-T2A-copGFP (CD521A-1, System Biosciences, Palo Alto, CA) was modified to replace EF1 with the rat neuron-specific synapsin 1 promoter (Syn) [21] to drive neuronal expression of either copGFP (LV-control) or human sAPPα [22] and copGFP, separated by a T2A cleavage signal (LV-sAPPα). Vectors were packaged in HEK293FT cells using a second-generation packaging system [23]. Viral particles were pseudotyped with either the vesicular stomatitis virus (VSVg) envelope, which has tropism for a wide variety of cells, but has limited spread from injection sites [24, 25] or a chimeric rabies/VSVg (RabB19) envelope (Addgene #88865) containing the SADB19 (B19) extra-virion and transmembrane domains and the intra-virion domain of VSVg, which by contrast can undergo retrograde transport [26]. Average viral genome titres, determined by quantitative RT-PCR [23], were 2 × 10^10^ and 2 × 10^9^ viral genomes/mL for VSVg and RabB19 pseudotyped LV, respectively.

### Cell culture methods for detection of expressed sAPPa

Primary neuronal mouse cultures were prepared from postnatal day 2 C57BL/6 mouse pups. Animals were deeply anaesthetized with pentobarbital (150 mg/kg, s.c.) and decapitated. After removing meninges and cerebellum from the brains, tissue was diced finely and then digested for 15 min at 37 °C on a MACS-Mix (Miltenyi Biotec, DE) in Leibovitz’s L-15 medium (Life Technologies, NZ) supplemented with 20 mM D-(+)-glucose (Sigma Aldrich, NZ), 0.8 mM kynurenic acid (Sigma Aldrich, NZ), 0.05 mM D(−)-2amino-5-phosphovaleric acid (AP5; Sigma Aldrich, NZ), 50 U/mL penicillin, 0.05 mg/mL streptomycin (penicillin-streptomycin; Life Technologies, NZ), 5.5 mM L-cysteine HCl (Sigma Aldrich, NZ), 12 U/ml Papain (Worthington Biochemical Corporation, NJ, US), 1 U/ml DNaseI (Life Technologies, NZ), 1.1 mM EDTA, 0.067 mM beta-mercaptoethanol, and 2% (*v*/v) B27 (Life Technologies, NZ). The enzymatic digest was stopped by blocking for 10 min at 37 °C on a MACS-Mix (Miltenyi Biotec, DE) in Leibovitz’s L-15 medium supplemented with 20 mM D-(+)-glucose, 0.8 mM kynurenic acid, 0.05 mM AP5, 50 U/mL penicillin, 0.05 mg/mL streptomycin, 10 mg/mL BSA and 10 mg/mL ovomucoid (Sigma Aldrich, NZ). Tissue was then triturated in OptiMEM supplemented with 20 mM D-(+)-glucose, 0.4 mM kynurenic acid, 0.025 mM AP5, 10 mg/mL BSA and 2% B27, passed through a 100 μm cell strainer and cells pelleted by centrifugation. The cell pellet was resuspended in culture media (Neurobasal A (Life Technologies, NZ) supplemented with 35 mM D-(+)-glucose, 0.4 mM L-glutamine (Life Technologies, NZ), penicillin (50 U/mL) and streptomycin (50 mg/mL), and 2% B27). Cells were plated at a density of 200,000 cells / well of a 24-well plate containing poly-L-lysine hydrochloride (Sigma Aldrich, NZ) coated coverslips. Cells were maintained in culture media in a 37 °C/5% CO_2_ incubator, with half of the volume replaced with fresh media every 3 days [27]. Cultures were transduced at 6 days in vitro (DIV) by adding 4 μl/well lentivirus expressing Syn.sAPPα-T2A-copGFP or Syn.T2A-copGFP, respectively. For immunocytochemistry, cells were fixed in 4% paraformaldehyde at 10 DIV and then stained with a MAP2 antibody (Millipore Cat# MAB3418 RRID:AB 11212326, 1:1000)/goat-anti-mouse Alexa488 (Cat# A-11001 RRID:AB_2534069, Life Technologies, NZ; 1:1000), and 4′, 6-diamindino-2phenylindole (DAPI; Life Technologies, NZ). For western blotting, media was replaced with culture media without B27 the day after transduction, and collected at 10 DIV.

### Stereotaxic surgery

At 4 or 10 months of age (prevention and rescue studies, respectively), animals were anaesthetised with a subcutaneous injection of ketamine/domitor/atropine (75/1/0.05 mg/kg body weight), and placed into a stereotaxic frame (Kopf Instruments; California, USA). Vectors were bilaterally injected through a 33 ga needle into the hippocampus using 2 μL of viral preparation per hemisphere at a rate of 150 nL/min. Four injection sites per hippocampus were used to optimize virus spread. Stereotaxic coordinates from bregma were (in mm): AP -1.8, ML ±1.2, DV -1.25 and − 1.95; and AP -2.5; ML ±1.8, DV -1.25 and − 1.95. The needle was left in place for 3 min after each injection before moving to the next site.

For surgeries at 4 months of age, a total of 25 WT animals were injected with LV-control, 16 Tg animals injected with LV-control, and 28 Tg animals injected with LV-sAPPα. For surgeries at 10 months of age, 26 mice were injected for electrophysiological analysis of LTP (9 WT with LV-control, 7 Tg with LV-control, and 10 Tg with LV- sAPPα).

### Behavioural testing

Behavioural testing commenced at 12 months of age, eight months after surgery in the 4 month age group. All behavioural testing and data analysis were conducted by an experimenter blind to the treatment conditions.

### Open field

The open field test was conducted in a 40 × 40 × 25 cm opaque white plastic box. The mouse was placed in the middle of the box and its behaviour observed and recorded for 5 min with a ceiling-mounted video camera linked to a computer running Ethovision XT7 software. The centre zone was defined as the 24 cm × 24 cm area in the middle of the open field and the percentage time spent in the periphery or the centre of the field was measured. At the end of the trial, the mouse was removed from the box, fecal boli were removed and the box cleaned with 10% ethanol.

### Morris water maze

The Morris watermaze testing was performed in a white plastic circular pool with a diameter of 100 cm and filled with water (20–22 °C) until 9 cm from the top. A small circular transparent Perspex platform (diameter 6 cm) stood 0.7 cm under the surface of the water and 21.5 cm from the pool wall. Prominent visible spatial cues with dissimilar features were located around the room at different heights. Performance was recorded using a ceiling mounted camera linked to the Ethovision XT7 program. Day 1 consisted of habituation by placing the mouse into the pool without the platform for 1 min. Days 2 and 3 comprised the cued learning phase, during which a visible flag was attached to the submerged platform (SE quadrant) and the mouse learned to seek out the platform. Each mouse underwent 6 trials/day with a maximum time in the pool of 60 s/trial and an inter-trial interval of 3 min. When the mouse reached the platform, it was allowed to remain on it for 15 s, and if the mouse did not reach the platform within 60 s, it was then gently placed on the platform and left there for 15 s.

The spatial reference memory acquisition phase was conducted on days 4–9, with 6 trials a day for 6 days, an inter-trial interval of 3 min, and the platform maintained in a fixed position different from during cued learning. The same platform was used for all sessions and each trial began from a different pseudo-randomly chosen start position with the mouse facing the wall. The mice were each allowed a maximum of 60 s in the pool, and if the mice did not arrive at the platform within the 60 s, they were then picked up and placed on the platform and allowed to remain there for 15 s. Total distance travelled (path length) and proximity data (calculated as the average distance from the platform during a trial and considered a sensitive measure of spatial learning [28, 29]), were measured.

Probe trials to test spatial reference memory were conducted just prior to training on the fourth day of reference memory acquisition (probe trial 1), and then 24 h after the last day of acquisition (probe trial 2). The mouse was placed in the pool for 60 s without the platform present and the number of platform crossings and proximity data were measured.

Immediately following probe trial 2, three days of spatial working memory testing were conducted. The experimental protocols were the same as for the reference memory acquisition testing except that the platform location was different for each day, although fixed for each day. The first day of testing was used for familiarizing the mice with the working memory task, and data were collected and analysed for the next two days of testing.

### Object recognition

Following a rest day, the mice were re-habituated to the open field box for 5 min. The object recognition task began the next day and consisted of placing two distinctly different objects in the centre of two adjacent quadrants of the box. The objects used (consisting of a plastic cube [4 × 4 × 4 cm], a cylinder [4 × 4 cm diameter], and a pyramid [4 × 4 × 4 cm] had 1.5 cm holes drilled into its sides in order to increase exploration [30]. The following day, one of the objects was replaced with a novel object with a different shape in order to test novel object recognition; 24 h later, the familiar object was moved to another quadrant of the box to test novel object recognition. The objects replaced and displaced were counterbalanced between mice. Each mouse was placed in the box and allowed to explore for 5 min. Exploring was defined as the mouse’s direct interaction with the object, such as nose and paw touching. Mice that did not achieve a total of 10 s of exploration within the given 5 min were excluded from the study. One WT-control, one Tg-control, and three Tg-sAPPα mice were excluded from the study based on these criteria. The trials were recorded by an overhead camera and mouse behaviour observed and analysed by the experimenter off-line. All objects and the exploration box were cleaned with 10% (*v*/v) ethanol solution between trials. For each object recognition task, the amount of time the animal spent exploring each object was measured. The data were then converted into a discrimination ratio, defined as:

$$ \frac{\mathrm{exploration}\ \mathrm{time}\ \mathrm{of}\ \mathrm{novel}\ \mathrm{object}\ \left(\mathrm{or}\ \mathrm{location}\right)-\mathrm{exploration}\ \mathrm{time}\ \mathrm{of}\ \mathrm{familiar}\ \mathrm{object}\ \left(\mathrm{or}\ \mathrm{location}\right)}{\mathrm{total}\ \mathrm{exploration}\ \mathrm{time}} $$

### Post-mortem tissue preparation

Beginning at least one week after the end of the behavioral testing, animals were deeply anaesthetized with pentobarbital (200 mg/kg, s.c.) and a transcardial perfusion was conducted with an ice-cold sucrose dissection solution (mM: 210 sucrose, 26 NaHCO_3_, 2.5 KCl, 1.25 NaH_2_PO_4_, 0.5 CaCl_2_, 3 MgCl_2_, 20 D-glucose) which had been bubbled with carbogen (95% O_2_–5% CO_2_). Following removal of the brain, one hemisphere was assigned for hippocampal slice electrophysiology and the other hemisphere for post-mortem analyses including western blots, ELISAs and histochemistry. The assigned hemisphere for each analysis alternated between left and right for successive mice.

### Extracellular electrophysiology

After removing the frontal cortex and cerebellum, the selected hemisphere was sectioned transversely into 400 μm coronal slices using a Leica vibrotome (VT 1000). Slices were transferred to a Millipore cell culture insert (Millicell®, Millipore, MA, USA) housed in a custom built incubation chamber containing artificial cerebrospinal fluid (ACSF, mM: 124 NaCl, 3.2 KCl, 1.25 NaH_2_PO_4_, 26 NaHCO_3_, 2.5 CaCl_2_, 1.3 MgCl_2_, 10 D-glucose) bubbled with carbogen. The slices were subsequently incubated at interface for 30 min at 32 °C and then at room temperature for at least 90 min. After this recovery period, slices were transferred to a recording chamber where they were gradually warmed to 32.5 °C while superfused (2 mL/min) with oxygenated (with carbogen) and humidified ACSF.

All recordings were made by an experimenter blind to the genotype and treatment condition of the mice. Field potentials were evoked using stimulating electrodes made from 50 μm Teflon-insulated tungsten monopolar electrodes placed in either the alveus or stratum radiatum and driven by custom constant-current stimulators controlled by custom Labview software. Evoked potentials were recorded using glass micropipettes (1.5–2.5 MΩ filled with ACSF), amplified (× 1000), filtered (0.3 Hz-3 kHz) and stored for later analysis using custom software. Population spikes were recorded in the stratum pyramidale in order to assess cell excitability across stimuli ranging from 10 to 200 μA (average of 3 responses at each stimulus intensity) to generate an input-output (I-O) curve, and then to assess recurrent inhibition by paired-pulse stimulation (PPI), where stimulation was first applied to the alveus to antidromically activate CA1 pyramidal cell axons (antidromic spike 75% of maximum amplitude), and then the stratum radiatum to evoke orthodromic population spikes (50% of maximum amplitude). Interpulse intervals ranged from 20 to 200 ms, with two pairs of stimuli at each pairing interval, followed by one orthodromic stimulus alone in association with each interval. PPI was expressed as the average of the two orthodromic responses for each pair at each interval divided by the average of all the orthodromic-only responses.

After PPI assessment, the recording electrode was moved to stratum radiatum where field excitatory postsynaptic potentials (fEPSPs) were recorded. Basal synaptic transmission was assessed by the input-output (I-O) measurements of fEPSPs by applying stimulation at increasing intensities as described above. Presynaptic paired-pulse facilitation (PPF) was tested by giving the slice three consecutive stimulations at interpulse intervals ranging from 20 to 200 ms. PPF was expressed as a ratio and was calculated as pulse 2 amplitude/pulse 1 amplitude. In LTP experiments, the stimulus current was set at a value that yielded half maximum fEPSP slope and the slice was stimulated every 30 s while a 30 min baseline was recorded. LTP was induced by giving either two (prevention study) or three (rescue study) theta-burst stimulation protocols (TBS) spaced 30 s apart. Each TBS protocol comprised 10 bursts at 5 Hz, with 5 pulses at 100 Hz per burst, at baseline stimulus intensity. After TBS, responses were recorded for a further 120 min. The initial slopes of the fEPSPs were measured, and each response expressed as a percentage change from baseline, which was defined as the average of the last 20 responses before TBS.

### Histochemical analysis

Coronal *s*ections (40 μm) from frozen tissue were mounted on slides and allowed to dry overnight. Congo-red was used to stain the sections to reveal amyloid plaques, with nuclei labelled with DAPI. Congo-red staining and DAPI were visualised on a Zeiss AX10 fluorescence microscope, attached to a Jenoptic camera and computer, and the percentage area covered by plaques was analysed using ImageJ. In short, images were converted to 8 bit, a threshold value was determined and maintained for all images, and the percentage area covered by plaques was calculated using the ImageJ algorithm.

### Western blots

The hippocampi not used for electrophysiology were snap-frozen on dry ice and stored at − 80 °C until protein extraction. Protein was extracted in solubilisation buffer (5 mM phosphate buffer pH 7.4, 0.32 M sucrose, 0.5 mM phenylmethylsulfonyl fluoride [PMSF in ethanol], 1 mM EGTA, 1 mM EDTA, and a protease inhibitor (cOmplete Ultra Mini Tablet, Roche)) without detergent, homogenized by pestle 30× and supernatant collected by two centrifugation steps at 14,000 *g* for 10 min and 30 min respectively at 4 °C. The resulting supernatant was identified as the soluble fraction. The resulting pellets were solubilized in a second buffer containing Triton-X and SDS (EGTA 1 mM, EDTA 1 mM, PMSF 0.5 mM, cOmplete protease inhibitor, Triton-X (1% *v*/v), sodium dodecyl sulphate (0.1% *w*/*v*) in phosphate buffered saline pH 7.4) and proteins solubilised by probe sonication (10 pulses at 1 s each; Qsonica, CT, USA). The resultant fraction was identified as the insoluble fraction. A DC protein assay (Bio-Rad) was use to quantify protein concentrations in both fractions.

Protein samples were separated on 9 or 12% (*w*/*v*) bis-acrylamide gels before transferring to a nitrocellulose membrane. Blots were incubated in Odyssey blocking buffer (LI-COR) at room temperature for 1 h. The primary antibody (microglia: Iba-1, WAKO 019–19,741, RRID:AB_839504; astrocytes: GFAP, Abcam-AB10062, RRID:AB_296804; presynaptic boutons: synaptophysin, Abcam-AB32127, RRID:AB_2286946; postsynaptic density: PSD-95, BD Transduction 610,496, RRID:AB_397862) or tubulin (Abcam-AB4074, RRID:AB_2288001) was prepared in phosphate buffered saline (PBS)-tween, 0.1% (w/v) BSA and 0.1% (v/v) NGS, overnight at 4 °C. The secondary antibody was either IRDye goat anti-rabbit680 or IRDye goat anti-mouse800 (LI-COR (1:10,000) in PBS/Tween), 1 h at room temperature. Blots were imaged on a LI-COR Odyssey imaging system, quantified using Image Studio 4 (LI-COR) after normalising to a loading control protein (tubulin).

Detection of sAPPα in the cell culture media was achieved by western blotting. Media was initially concentrated by ammonium sulfate precipitation (at 75% saturation). Proteins were then separated on a 10% (w/v) SDS-PAGE gel and transferred to a nitrocellulose membrane (100 V, 1 h). Blocking overnight in 1% (w/v) milk powder-PBS tween was followed by incubation for 2 h at room temperature with an N-terminal APP antibody (Cat# A8967 RRID:AB_258427, Sigma Aldrich, NZ; 1:1000), diluted in blocking solution (1% milk powder-PBS tween).. After three washes in PBS-0.3% Tween-20 (PBS-T), anti-rabbit-HRP secondary antibody (Cat# NA934, RRID:AB_772206, GE Healthcare Life Sciences) was applied for 2 h at room temperature (1:10,000 in PBS-T). Unbound secondary antibody was removed with three PBS-T washes and the blot was developed using Amersham ECL Prime Western Blotting Detection Reagent (GE Healthcare Life Sciences) and imaged using a Fuji LAS-3000 ECL imaging system.

### Enzyme-linked immunosorbent assay (ELISA)

Aβ and sAPPα concentrations of the hippocampal samples were measured using four ELISA kits: Human amyloid β (1–42) Assay Kit (IBL, Hokkaido, Japan, 27,711), human amyloid β (1–40) Assay Kit (IBL, 27,713), human sAPPα high sensitive ELISA (IBL, JP27734), and Mouse/Rat sAPPα (highly sensitive) ELISA (IBL, JP27419). The procedures were performed according to the kit instructions. ELISA for mouse and human sAPPα were performed on the soluble fraction (as prepared for western blotting), and ELISA for human Aβ (1–42) and (1–40) were performed in both the soluble and insoluble fractions. Despite its ability to detect recombinant human sAPPα samples, the human sAPPα kit was not able to detect either native or virus-mediated sAPPα expression in the Tg mice, and thus we could not determine degree of up-regulation of sAPPα levels in the tissue. Therefore copGFP expression was used as the marker of successful transduction in the hippocampus.

### Statistical analysis

Behavioural and electrophysiological statistical data were calculated in Microsoft Excel and SPSS v21 (IBM), and differences between groups were compared using one-way analysis of variance (ANOVA) or a mixed model two-way ANOVA with repeated measures on one factor with Lower-Bound corrected values. Post-hoc tests were conducted using Tukey’s test, with significance set at *p* < 0.05. All group data are presented as mean ± SEM. Planned comparisons were conducted using Students t-tests comparing WT-control group with Tg-control group to examine genotype, and Tg-control group with Tg-sAPPα group to determine treatment effect. T-tests were conducted using SPSS version 21 software.

## Results

### Expression of sAPPα

**Fig. 1:**
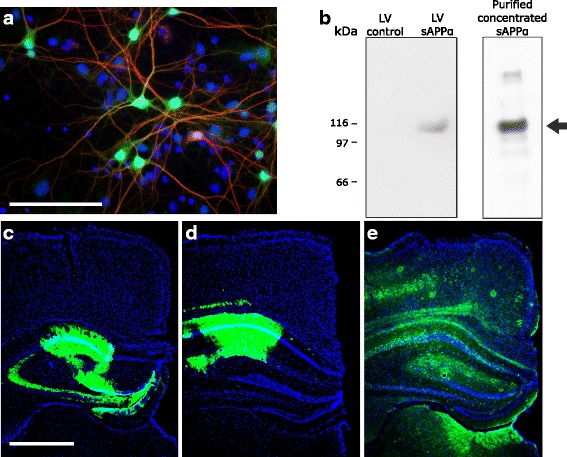
In vitro and in vivo lentiviral transduction of hippocampal neurons with sAPPα-copGFP. **a** The transduction marker copGFP (green) was expressed in mouse primary hippocampal neurons co-labelled with the neuronal marker MAP2 (red) and the nuclear marker DAPI (blue). Scale bar, 50 μm. **b** Detection of sAPPα in media from primary mouse neural cultures transduced with LV-sAPPα, but not LV-control. The right-hand lane of the western blot illustrates the band from a sample fortified with purified sAPPα [22]. **c**-**e** Examples of in vivo copGFP expression (green) showing transduction of hippocampal regions containing LV-transduced neurons in slices from wild-type (**c**), transgenic-control (**d**) and transgenic-sAPPα (**e**) mice. Scale bar: 1 mm

In order to test whether transduction of cells with LV-sAPPα resulted in the expression from the viral vector and secretion of sAPPα, primary mouse neural cultures were transduced with LV-sAPPα or LV-control. GFP expression was localised to neurons as expected (Fig. 1a), whereas sAPPα was specifically secreted into the media from the transduced cultures (Fig. 1b). The persistence of cell transduction in vivo was assessed by expression of copGFP histologically, which was used as a surrogate marker given that the ELISA kit was not able to determine sAPPα levels per se. Ongoing expression was evident in area CA1 and often the dentate gyrus in animals even 10 months after surgery (Fig. 1c-e). Although the intensity of copGFP fluorescence was reduced in the Tg-sAPPα animals compared to the two other groups that received the copGFP-only vector, clear transduction in the CA1 pyramidal cell layer and neuropil was nonetheless still visible, as well as in the dentate gyrus and overlying neocortex in some animals. No animals were excluded from the study due to a lack of copGFP expression in the hippocampus, as observed in the post-mortem analysis.

### Prevention study

In order to test whether human sAPPα expressed chronically from an early presymptomatic stage can act to delay or prevent the development of AD-like symptoms, we studied Tg mice injected with LV-sAPPα at 4 months of age, with behavioural testing commencing at 12 months of age. Control groups were Tg and WT mice injected with the LV-control virus. Because there were no significant differences in the LTP and reference memory data between the VSVg and rabies packaged sAPPα constructs, all data from the two packaging procedures were pooled in the analysis below.

### Behavioural testing

**Fig. 2:**
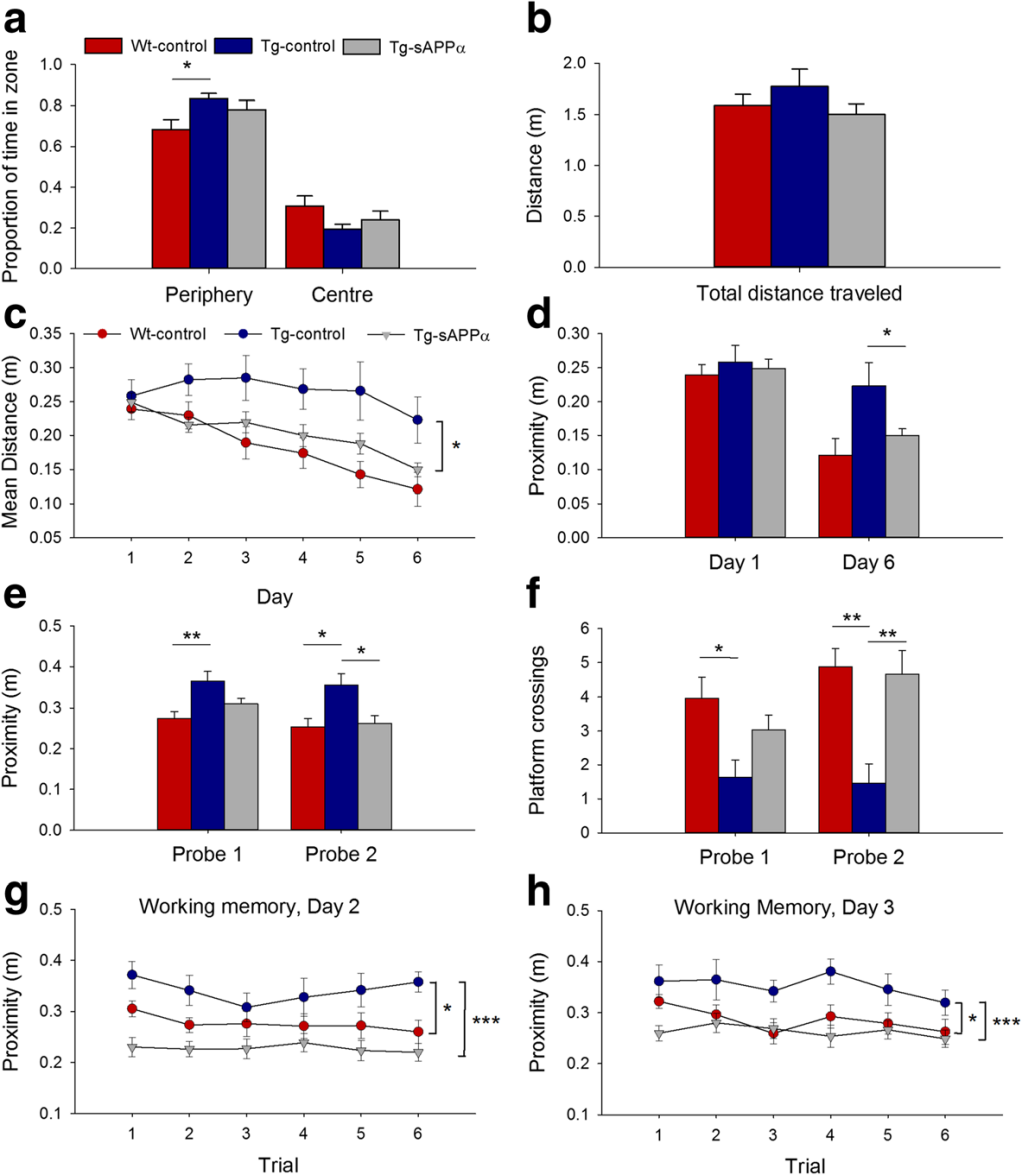
sAPPα transduction rescued spatial learning and memory in the APP/PS1 mice. **a** In the open field all groups preferred the periphery (*p* < 0.001), but this preference was stronger in Tg-controls (*n* = 11) compared to WT-control (*n* = 22, *p* < 0.05, Students t-test). The Tg-sAPPa group showed a trend toward reduced time in the periphery (*n* = 23, *p* = 0.084) compared to Tg-controls. **b** Mean distances travelled by each group were not significantly different. **c** For spatial reference memory acquisition in the water maze, there was a significant group main effect (F_(1,54)_ = 3.33, *p* = 0.043) for proximity to the platform, with a strong trend for Tg-controls to be impaired relative to WT-control animals (Tukey *p* = 0.071), while Tg-sAPPα animals performed significantly better than Tg-control animals (*p* = 0.046). **d** There was a significant group main effect (F_(1,54)_ = 3.33, p = 0.043) with a strong trend for impaired performance on day 6 by the Tg-control group compared to WT-controls (*p* = 0.068) and by enhanced performance of the Tg-sAPPα group compared to Tg-controls (*p* = 0.018). **e**, **f** Tg-control mice had significantly poorer memory for the platform position than WT-controls (Probe 1: proximity *p* = 0.004, crossings *p* = 0.037; Probe 2: proximity *p* = 0.016, crossings *p* = 0.006). Tg-sAPPα mice exhibited a partial rescue in Probe 1 and a complete rescue in Probe 2 (proximity *p* = 0.022, crossings *p* = 0.006 compared to Tg-control). **g**, **h** Spatial working memory testing revealed a significant group main effect (G, Day 2: *p* < 0.001; H, Day 3: *p* = 0.004). On both days, Tg-controls performed significantly worse than WT-controls (Day 1: *p* = 0.025; Day 2: *p* = 0.034) while Tg-sAPPα animals were significantly better than Tg-controls (Day 1: *p* < 0.001; Day 2: *p* = 0.003). *p < 0.05, **p < 0.01, ***p < 0.001

In the open field test of exploration, animals in all groups generally spent more time in the periphery of the open field, compared to the centre. Tg-control animals spent significantly more time in the periphery of the open field compared to WT-control animals, suggesting a modest increase in anxiety in the Tg-control group. There was a trend for this genotype effect to be reversed in the Tg-sAPPα group compared to Tg-control animals (*p* = 0.084), such that there was no significant difference between the Tg-sAPPα group and the WT-controls (Fig. 2a). There was no difference in distance travelled between groups (Fig. 2b).

### Water maze cue learning

There were no differences between groups in learning the cued phase of the water maze test (data not shown). However, based on the observations noted in this phase, two animals, one from the Tg-sAPPα group and one from the Tg-control group, were removed due to constantly swimming around the periphery of the water maze pool and thus not learning to use the platform as a goal.

### Water maze spatial reference memory task

In the acquisition phase of the water maze spatial reference memory task, a mixed-model two-way ANOVA revealed a significant main effect of training days for the path-length taken to reach the platform, as well as for proximity, i.e. average distance from the platform. There was also a significant main effect of treatment group on proximity to the platform with Tukey post-hoc tests revealing a strong trend for an impairment in the Tg-control group relative to the WT-control group, and a significant enhancement of performance back to WT levels for the Tg-sAPPα group, compared to the Tg-control group (Fig. 2c, d). For the first probe trial (24 h after the first three days of training), there was a significant effect of group on proximity, with the Tg-control being impaired relative to the WT-control group (*p* = 0.004), with a partial rescue for the Tg-sAPPα group. By the second probe trial, there was a complete and significant rescue of memory in the Tg-sAPPα group compared to Tg-controls (F_(2,56)_ = 4.69, *p* = 0.013; Tukey’s *p* = 0.022; Fig. 2e). The results were similar for platform crossings, where the Tg-control group showed fewer platform crossings than the WT-control group during probe trial 1 (*p* = 0.037), but no significant rescue for the Tg-sAPPα group (*p* = 0.182). However, by probe trial 2, there was both a genotype effect (*p* = 0.006), and a treatment difference such that the Tg-sAPPα group showed significantly more crossings relative to the Tg-control group (*p* = 0.006), back to the level of the WT-control group (Fig. 2f).

### Spatial working memory

The last three days of the water maze testing were used for working memory testing. The first day’s training was used to teach the animals the new task. On each of days 2 and 3, there was a significant main effect of treatment group, whereby the Tg-controls performed more poorly than the WT-controls and the Tg-sAPPα group showed a virtually complete rescue of working memory performance to wildtype levels (Fig. 2g, h).

To summarize, the impairment in water maze learning by the Tg-control animals was completely prevented by sAPPα expression. Spatial memory retention, as evidenced during the probe trials, showed the same effects. Tg-control mice were also impaired on the spatial working memory task, and this was again prevented by sAPPα expression. Thus virus-mediated sAPPα expression was an effective treatment for both reference and working spatial memory tasks, even when commenced 8 months prior to behavioural testing.

### Synaptic transmission and plasticity

**Fig. 3:**
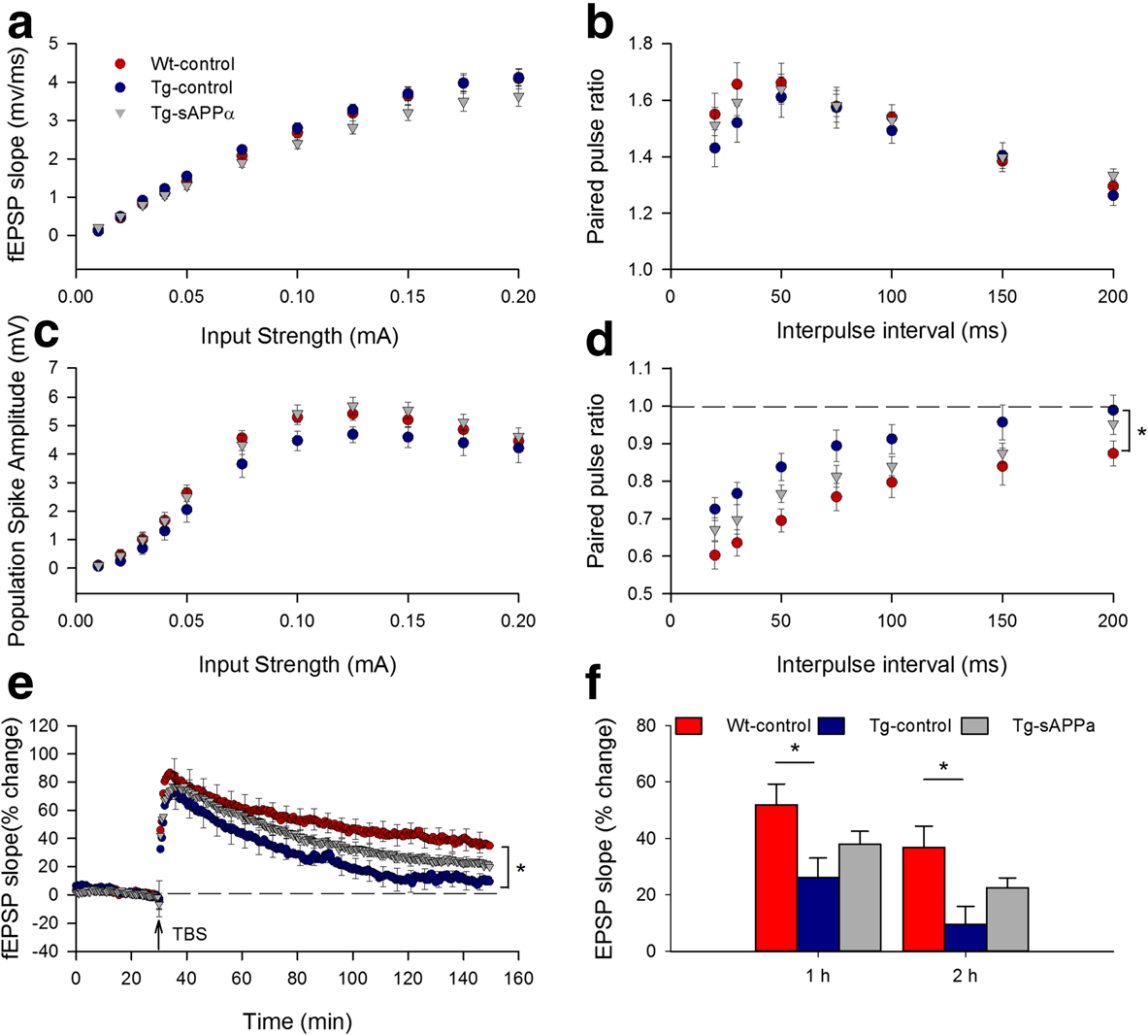
Expression of sAPPα partially restored hippocampal LTP in transgenic mice. **a**, **b** There were no significant genotype or treatment effects on the I/O curves in CA1 for either the (**a**) EPSP slope, (*p* = 0.17 and 0.14, respectively) or (**b**) population spike amplitude (*p* = 0.14 and 0.63, respectively, indicating that basal synaptic transmission and cell excitability were unaffected in Tg mice and sAPPα over-expression. **c** There were no group differences in paired-pulse facilitation of the fEPSP (WT-control: n = 22; Tg-control: *n* = 12; Tg-sAPPα: *n* = 17, F_(2,48)_ = 0.32, *p* = 0.73), nor a group x interval interaction (F_(2,48)_ = 0.96, *p* = 0.39), indicating no difference in basal transmitter release probability. (**d**) Paired-pulse inhibition was reduced in Tg-controls compared to WT-controls (WT-control: *n* = 16; Tg-control: *n* = 10; F_(2,38)_ = 4.18, *p* = 0.023), indicating an impairment of recurrent inhibition. A partial rescue was produced by sAPPα expression. (**e**, **f**) TBS delivered to the Schaffer collaterals induced robust potentiation in all groups but which decayed at different rates between groups. Significant group differences were observed at both 1 h (F_(2,49)_ = 4.20, *p* = 0.021) and 2 h post-TBS (F_(2,49)_ = 5.44, *p* = 0.007) with Tg-controls significantly impaired (26.1 ± 6.9%, *n* = 11) compared to WT-controls (51.9 ± 7.4%, *n* = 21; *p* < 0.05) after 1 h. LTP in the Tg-sAPPα indicating a partial recovery of LTP expression. The partial recovery was still observed 2 h post-TBS (WT-controls: 36.8 ± 7.5%, *n* = 21; Tg-controls: 9.7 ± 6.2%, *n* = 11; Tg-sAPPα: 22.5 ± 3.5%, *n* = 18). *p < 0.05

To test for genotype and sAPPα effects on hippocampal electrophysiology in the same animals, we first undertook I/O curve and paired-pulse analyses of the Schaffer collateral input to area CA1, beginning at least one week after the end of behavioural testing. Mixed model ANOVA revealed that there was no main effect of group on the fEPSP initial slope (*p* = 0.17), nor a group x stimulus interaction (*p* = 0.12; Fig. 3a). Similarly, there was no group effect on population spike amplitude (*p* = 0.14, ns) nor group x stimulus interaction (*p* = 0.63; Fig. 3b). These data indicate that basal synaptic transmission and cell excitability were not affected by either the Tg genotype or the sAPPα (and copGFP) expression.

Paired-pulse stimulation was used to test for genotype or treatment effects on short-term plasticity of excitatory synaptic transmission. As expected, mixed-model ANOVA showed a main effect of inter-pulse interval (F_(1,48)_ = 48.66, *p* < 0.001), with longer intervals associated with less paired-pulse facilitation. However, there was no main effect of group (Fig. 3c), nor group x interval interaction. Thus there was no effect of viral transduction on short-term presynaptic plasticity mechanisms. To test for the strength of recurrent inhibition, a conditioning pulse was first delivered to the alveus in order to antidromically activate CA1 axons, and thus generate recurrent excitation of inhibitory interneurons, prior to a test pulse to the Schaffer collateral afferents that was above population spike threshold. Mixed-model ANOVA revealed a main effect of group (Fig. 3d), with Tg-controls showing less paired-pulse inhibition than WT-controls. The Tg-sAPPα group was not significantly different from either group, indicating a partial rescue of recurrent inhibition by sAPPα expression.

### Long-term potentiation

Following theta-burst stimulation, all three groups showed a large initial potentiation that steadily decreased but without returning to baseline over the ensuing two hours (Fig. 3e). One-way ANOVAs revealed significant group differences at both 1 h and 2 h post-TBS (Fig. 3f). At 1 h, Tg-controls showed significantly impaired LTP compared to WT-controls. The LTP for the Tg-sAPPα group occurred at an intermediate level that was not significantly different from either of the other groups, indicating a partial amelioration of the genotype effect on LTP. A similar pattern of results was found for the data at the 2 h time-point (Fig. 3f).

### Amyloid-β load

**Fig. 4:**
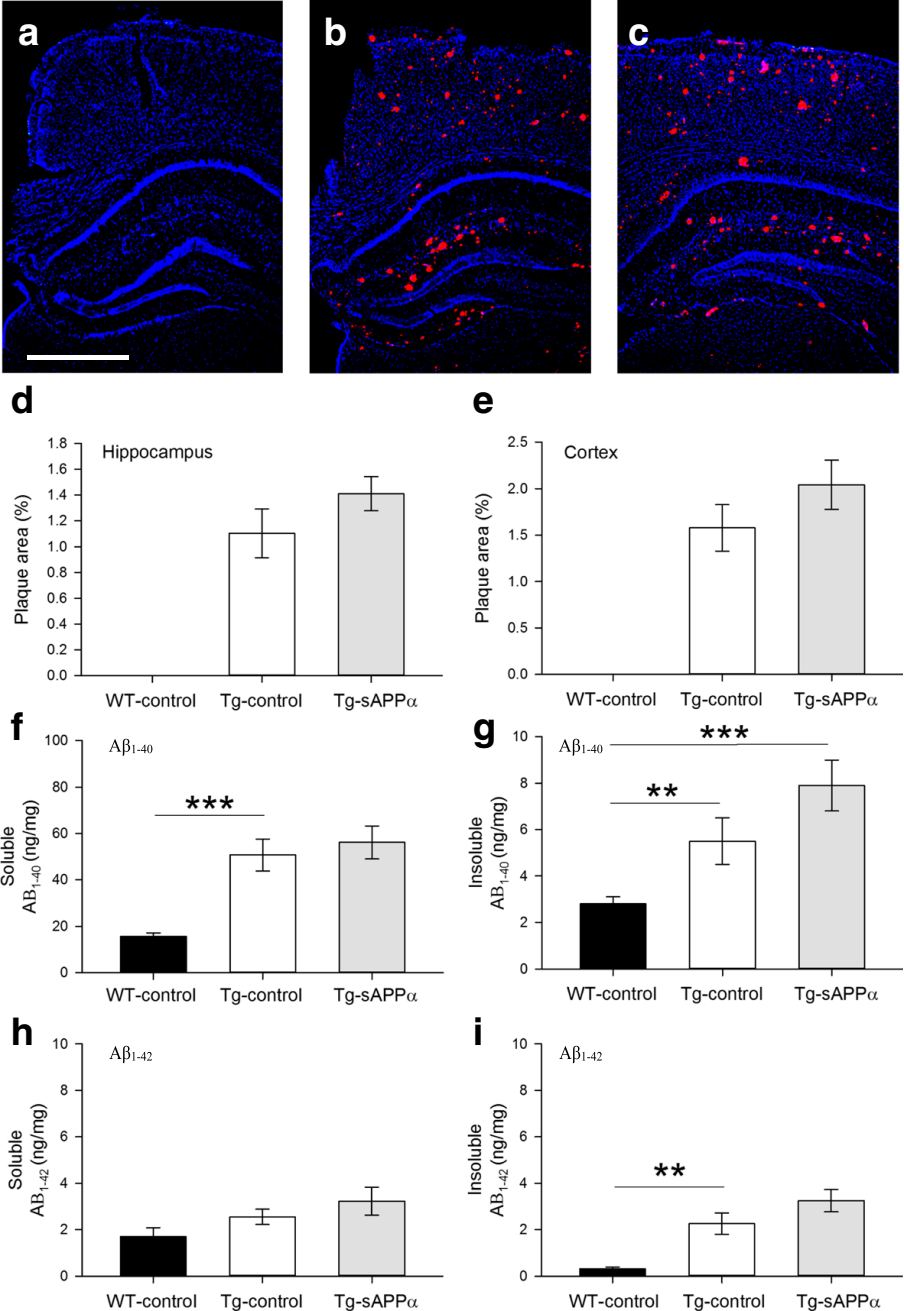
Effect of sAPPα expression on the development of amyloid pathology. **a-c** Congo red staining of coronal brain sections from (**a**) WT-control, (**b**) Tg-sAPPα and (**c**) Tg-control animals revealed an absence of amyloid plaques in WT-control brains, but extensive plaque formation in Tg-sAPPα and Tg-control brains. **d-e** No significant differences in plaque density were observed between Tg- sAPPα and Tg-control in the hippocampus (*p* = 0.316, **d**) or overlying neocortex (*p* = 0.297, E). **f-i** Human amyloid-β load in the hippocampus. **f** Soluble Aβ1–40 levels differed between groups (one-way ANOVA F(2,27) = 17.40, *p* < 0.001), with higher levels in in Tg-control animals compared to wildtypes (WT-control: 15.6 ± 1.6 ng/mg, *n* = 12; Tg-control: 50.7 ± 6.9 ng/mg, *n* = 6, post-hoc Tukey *p* = 0.001), but no effect of sAPPα treatment compared to Tg-control (Tg-sAPPα: 56.1 ± 7.1 ng/mg, n = 12, Tukey = 0.820). **g** Insoluble Aβ1–40 levels showed a significant overall group effect (F(2,28 = 10.70) and while there was no significant difference between WT-control and Tg-control (WT-control, 2.8 ± 0.3 ng/mg, *n* = 13, Tg-control, 5.5 ± 1.0 ng/mg, *n* = 6, Tukey *p* = 0.145), the levels were elevated in the Tg-sAPPα group compared to WT-control (Tg-sAPPα 7.9 ± 1.1 ng/mg, Tukey p < 0.001) and they were not different to Tg-control (Tukey p + 0.194). (H) Soluble Aβ1–42 levels did not show any differences between groups. **i** For the insoluble fraction, there was an overall group effect (F(2,28) = 20.47, p < 0.001, whereby Tg-controls showed the expected greater Aβ1–42 load (2.26 ± 0.46 ng/mg, *n* = 6) compared to WT-controls (0.31 ± 0.077 ng/mg, *n* = 13; Tukey p = 0.006) and this level was not significantly affected by sAPPα over-expression (3.25 ± 0.48 ng/mg, *n* = 12, Tukey *p* = 0.220). **p < 0.01, ***p < 0.001, Scale bar: 1 mm

To examine the effects of LV-sAPPα expression on amyloid-β load in the Tg mice, we examined a selection of hemispheres opposite to those used for the electrophysiology and stained with Congo red. There were no plaques observed in the hippocampus or overlying cortex of WT-control animals (Fig. 4a**)**. In the Tg mice, there were large numbers of plaques, but no significant difference in the area of stained plaques between the Tg-sAPPα and Tg-control animals for either the hippocampus or the overlying cortex (Fig. 4b-e).

Human amyloid-β load was also assessed by ELISA for both soluble and insoluble fractions made from the hippocampus. A one-way ANOVA revealed an overall effect of group for soluble Aβ1–40 (*p* < 0.001), with a clear increase in the Tg-control animals compared to wild-type animals (post-hoc Tukey *p* = 0.001), but no effect on this elevated level by the expression of sAPPα (*p* = 0.820 compared to Tg-control; Fig. 4f). Aβ1–40 in the insoluble fraction showed a similar pattern of effects (ANOVA p < 0.001; Fig. 4g). Aβ1–42 levels in the insoluble fraction showed an overall effect of group (ANOVA p < 0.001) with Tg-controls showing the expected greater Aβ1–42 load compared to WT-controls (Tukey *p* = 0.006). Once again there was no significant effect of sAPPα expression on this elevated level (Tukey *p* = 0.296 compared to WT-control). A similar pattern of results was seen for soluble Aβ1–42 (Fig. 4h, i). Thus, the above-mentioned rescue of spatial memory and partial restoration of LTP was achieved in the absence of affecting amyloid load.

### Western blot results

**Fig. 5:**
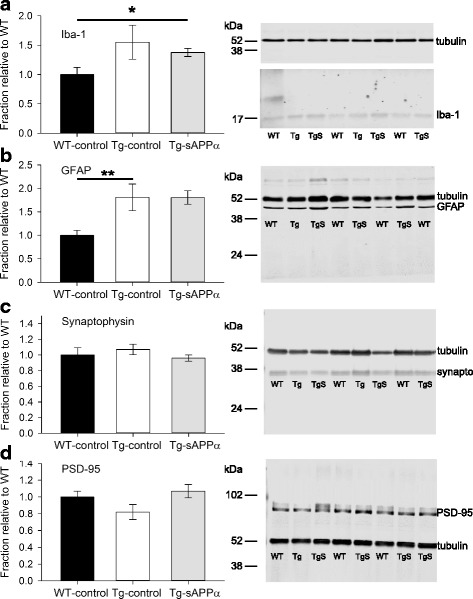
Cellular markers for neuronal and non-neuronal cells. **a** Western blot analysis of the microglial marker Iba-1 (insoluble fraction), normalised to WT-control, showed an overall group effect (one-way ANOVA F(2,28) = *p* = 0.041) with a trend toward higher levels in Tg mice (WT-control: 1.0 ± 0.083, n = 12; Tg-control: 1.37 ± 0.299, n = 6), that was even more evident in the Tg-sAPPα group (Tg-sAPPα: 1.39 ± 0.065, *n* = 13; post-hoc Tukey *p* = 0.046). **b** GFAP levels (insoluble fraction) showed a significant overall group effect (F(2,27) = 10.92, *p* < 0.001), whereby there was significantly higher levels in the Tg-control group compared to the WT-control group (WT-control: 0.998 ± 0.105, *n* = 13; Tg-control: 1.81 ± 0.284, *n* = 6, Tukey *p* = 0.007) and expression of sAPPα did not alter this higher expression level (Tg-sAPPα: 1.86 ± 0.23, *n* = 11, Tukey *p* = 0.974). **c** Neither genotype (WT-control: 1 ± 0.02, n = 13; Tg-control: 1.07 ± 0.674, *n* = 6) nor sAPPα treatment (Tg-sAPPα: 0.96 ± 0.043, n = 12) affected the presynaptic marker synaptophysin (soluble fraction). **d** Levels of the postsynaptic marker PSD-95 (insoluble fraction) were also not affected by the APP/PS1 genotype (WT-control: 1.00 ± 0.072, *n* = 13; Tg-control: 0.82 ± 0.086, *n* = 5), although the PSD-95 levels tended to be higher for the Tg-sAPPα group compared to Tg-controls (Tg- sAPPα: 1.066 ± 0.082, *n* = 12, t_(17)_ = − 1.90, *p* = 0.074. Representative western blots are presented. Note that in the case of Iba-1, its illustration and that of tubulin are from the same blot, but at different exposures. Tubulin was used as the loading control. *p < 0.05, **p < 0.01; synapto: synaptophysin; Lane labels: WT, WT-control; Tg, Tg-control; TgS, Tg-sAPPα

To assess the effect of genotype and sAPPα expression on inflammatory markers for microglia and astrocytes, we used western blot to determine the levels of markers for these cells, Iba-1 and GFAP, respectively, in the hippocampus. A one-way ANOVA comparing hippocampal Iba-1 levels between groups, normalised to WT-control, showed a significant effect of group (*p* = 0.041), indicating increased Iba1 in the transgenic mice but no significant difference between the Tg-control and the Tg-sAPPα groups (post-hoc Tukey *p* = 0.991; Fig. 5a). GFAP levels were also significantly different between groups (*p* < 0.001) with significantly higher levels in the Tg-control group compared to the WT group (*p* = 0.007) but once again this difference was also seen in the Tg-sAPPα group (*p* = 0.001; Fig. 5b).

To determine whether the occurrence of synaptic contacts was affected by genotype or sAPPα expression, we tested for expression of the presynaptic and postsynaptic proteins synaptophysin and PSD-95, respectively, in the hippocampus. There was no significant effect of group on the levels of presynaptic marker synaptophysin (Fig. 5c). There was also no group effect on the levels of the postsynaptic marker PSD-95 (Fig. 5d). The general lack of change in these synaptic protein markers was consistent with the lack of effects on the fEPSP I-O curve (cf. Fig. 3a).

### Rescue of LTP deficits by LV-administered sAPPα

**Fig. 6:**
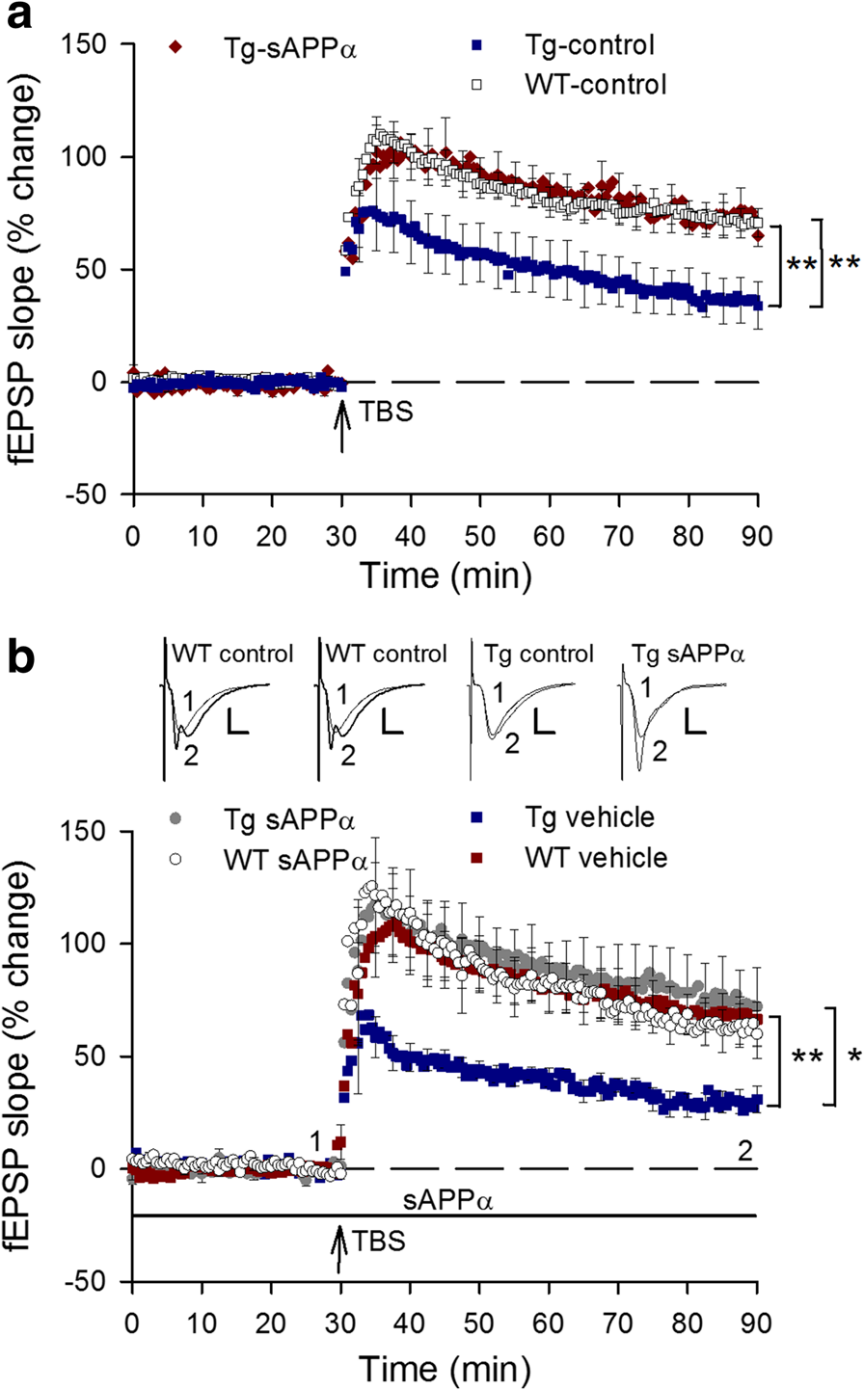
Delivery of sAPPα, either in vivo or in vitro, after plaque development, completely rescued the impaired LTP in Tg-controls. **a** Chronically administered sAPPα by lentivirus-mediated expression in adult (10 months) Tg mice completely rescued the deficit in hippocampal LTP, measured 3 months after viral transduction (at 13 months of age). LTP measured 60 min after TBS (arrow) revealed a significant deficit in LTP expression in Tg-control mice compared to WT-controls (WT-controls: 71.1 ± 6.7%, *n* = 11; Tg-controls 36.3 ± 10.0%, *n* = 9; *p* = 0.008). This deficit was completely rescued by sAPPα over-expression (Tg-sAPPα: 72.2 ± 2.4%, *n* = 12; *p* = 0.008 compared to Tg-controls). **b** LTP induced in hippocampal slices from aged Tg mice (18–20 months of age), was impaired compared to WT-controls when measured 60 min post-TBS (WT-control: 67.2 ± 7.5%, *n* = 9; Tg-control: 29.4 ± 4.7%, *n* = 7; *p* = 0.002). LTP expression was again completely rescued by acutely applied recombinant human sAPPα (10 nM) beginning 30 min before delivery of the TBS (Tg-sAPPα: 73.7 ± 16.7%, *n* = 10; *p* = 0.737 compared to WT-controls, *p* = 0.048 compared to Tg-controls). No effect of sAPPα on WT-control LTP was observed (WT-sAPPα: 62.1 ± 9.4%, n = 6; *p* = 0.684)

To address whether lentivirus-mediated expression of human sAPPα could also rescue hippocampal synaptic plasticity *after* plaque formation, a separate group of animals was transduced at 10 months of age, prior to in vitro electrophysiology beginning at ~ 13 months of age (the same age as for the prevention study). Characterization of the I-O curves for the fEPSP and population spike (data not shown) again revealed no main effect of treatment group, indicating that neither genotype nor sAPPα treatment affected basal synaptic transmission or cell excitability. Paired-pulse facilitation of the fEPSP was likewise unaffected (data not shown). In contrast, LTP was significantly impaired in the Tg-control group compared to the WT-control group, as described for the prevention study (cf. Fig. 3e, f). Notably, LTP was fully restored to control levels in the Tg-sAPPα group in this experiment (Fig. 6a).

### Rescue of LTP deficits by acutely administered sAPPα

Finally, we asked whether even acute administration of sAPPα to the bathing medium would be sufficient to rescue LTP in slices prepared from Tg mice (18–20 months of age). Administration of recombinant human sAPPα_1–612_ (10 nM, [22] to the bathing solution 30 min prior to TBS caused no change in fEPSP slope as assessed by I-O curves for either genotype and no change to the baseline responses (data not shown). LTP induced by TBS was again significantly impaired in Tg slices compared to WT slices (Fig. 6b). Bath administration of sAPPα prior to and throughout the LTP protocol led to a complete rescue of LTP in Tg slices, without affecting LTP in the control slices. These data show that LTP is sensitive to the level of sAPPα available around the time of induction, and that long-term delivery is not required for LTP deficits to be reversed in this animal model of AD. It remains to be determined whether the concentration of sAPPα becomes critical for LTP just before, during or just after the induction protocol.

## Discussion

Our study has provided the first evidence that long-term expression (> 8 months) of the human form of sAPPα, beginning before development of the disease phenotype, can substantially mitigate the development of cognitive and synaptic deficits in a mouse model of AD. We observed that such treatment offered complete protection of spatial and working memory as measured by water maze performance 8 months after transduction. We further observed an apparent partial prevention of the deficit in LTP measured 9–10 months post-transduction that may in turn have contributed to the behavioural effect. Although the virus transductions targeted the hippocampus, expression in the overlying sensorimotor cortex was evidenced in at least some of the cases, and thus this brain region may have also contributed to the behavioural rescue. Collectively, these findings suggest that lasting restoration of sAPPα production beginning early in the disease process may be efficacious in preventing or at least delaying the later expression of the range of deficits that typically define the disease.

Given its neuroprotective, neurotrophic and plasticity enhancing properties, the therapeutic potential of sAPPα has been gaining considerable interest in recent years, particularly with respect to Alzheimer’s disease but also for other neurological disorders [16, 18, 31, 32]. Indeed, acute intracerebroventricular administration of sAPPα shortly after traumatic head injury improved structural and functional outcomes in both normal [33] and APP knock-out mice [17]. Acute administration has also been used to ameliorate symptoms associated with ischemic brain injury [19] and normal aging [34, 35]. However, repeated acute administration of sAPPα would be problematic for AD patients, especially if intracerebral methods were to be required. Methods that engender longer term sAPPα up-regulation should therefore be advantageous. Recently, expression of mouse sAPPα in the hippocampus using a different viral vector (AAV), beginning well after strong phenotypic expression of AD-like symptoms, was found to rescue spatial memory and, as we observed, an associated substantial but not complete rescue of LTP measured several months after transduction [20]. Our study replicated the rescue of LTP using viral transduction of sAPPα that commenced at 10 months of age, even though a different viral vector (lentivirus), and a different sAPPα construct (human sAPPα) was used. Such studies show the effectiveness of sAPPα administration after disease onset. Importantly, we found that the same transduction procedure at 4 months of age rendered a long-term benefit lasting at least 9–10 months in protecting memory and LTP. The time of injection together with its long-lasting benefits suggests that, once predictive biomarkers for AD become readily available, a sAPPα-based therapy may be useful even in preventing or delaying the earliest onset of the disease, before individuals develop clinical symptoms. Together these studies make a strong statement about the therapeutic potential of sAPPα in AD.

### Behavioural improvement in the absence of amyloid load reduction

It was notable in the present study that the memory and LTP improvements occurred in the absence of a decrease in amyloid load, as measured by either Congo red staining of plaques or by ELISA. This contrasts with the previous AAV study in which a partial reduction in amyloid levels and plaque load was evident [20]. The reasons for this difference are not clear, but may relate to differences in the expression system and thus level of sAPPα expression, or the difference in timing of the transduction event. However, in accord with the present study, a number of treatment approaches have generated improvements in cognitive performance but not Aβ pathology [36–40]. Interestingly, this could occur even with animals immunized with an Aβ peptide or an antibody against Aβ. These results may reflect the high degree of plaque load in some of the animal models used, including the APP/PS1 model that we used.

Because of the lack of effect by sAPPα on amyloid load in the present study, the effectiveness of sAPPα expression would appear to be due to increasing the functional capabilities of the diseased brain, rather than modifying the disease process per se, although this latter point merits further investigation. Therefore, the effect of sAPPα in our study could be characterized as increasing cognitive reserve, allowing the brain to function more normally despite the presence of clear pathology. Cognitive reserve has been hypothesized to account for the greater than expected functioning of humans despite a high plaque load as determined, for example, by positron emission tomography [41, 42]. Fol et al. [20] also showed changes in spine density supportive of neuronal plasticity and correlating with the LTP findings. We have not explored these local changes in spine densities, but did not see global increases in pre- or postsynaptic markers by western blot.

### Immediacy of sAPPα’s effects

It is clear from the studies cited above and the present experiments, that sAPPα can exert powerful effects on brain function and neural plasticity, whether administered acutely or by expression over extended periods of time. Insofar as its effects in AD models may not be due to modifying plaque pathology per se, the question can be asked whether long-term administration over months (whether delivered early or late in the disease process) is needed to cause LTP and memory rescue, or whether even acute administration can be effective in AD models, as shown in a study of aging-related memory decline [35]. Accordingly, we administered sAPPα to hippocampal slices from 18 to 20 mo APP/PS1 and wild-type mice and investigated its effect on LTP as induced by a strong TBS protocol. Interestingly, sAPPα completely rescued LTP induction and persistence in the transgenic mice, showing that raising sAPPα levels even acutely might have benefits for AD patients, albeit in a transient manner. The rescue was equally strong when viral transduction commenced just 3 months before the start of LTP testing. The reason for the differential effects on LTP in the prevention versus rescue experiments is not clear, although it may relate to the greater number of TBS trains used in the rescue experiments, which gave a larger and longer lasting form of LTP that may depend more on the protein synthesis mechanisms that sAPPα can trigger [12]. sAPPα administration did not however increase the already strong LTP exhibited by wild-type slices. This finding is consistent with our previous work in the dentate gyrus in vivo, where only a small improvement in a strongly induced control LTP could be elicited [10]. Thus, sAPPα appears to work optimally under conditions of either weakly induced LTP or LTP impairment, as suggested previously [10, 11, 18].

### Mechanisms of sAPPα action

The mechanisms by which sAPPα enhances neural function in either AD models or normal animals are not clear. sAPPα is known to activate a number of signalling cascades involving mitogen-activated protein kinase, tyrosine kinases, cyclic GMP and protein kinase G (PKG), nuclear factor kappa-light-chain-enhancer of activated B cells (NFκB), PI3 kinase, and calcium/calmodulin-dependent protein kinase II (reviewed in [43, 44]. Downstream effects include increased gene expression [6, 45] and protein synthesis [12]. However, which if any of these cascades might be involved in the LTP rescue remains to be determined, although the activation of PKG has been suggested as one important signal transduction pathway for this purpose [9].

## Conclusion

In summary, we have provided evidence that expression of human sAPPα in the mouse hippocampus can not only prevent the development of an AD-like phenotype, but also rescue synaptic plasticity once the phenotype has developed. Whether a sAPP-based gene therapy will be a viable treatment option for AD remains to be seen, although gene therapy trials have commenced for other therapeutic agents in AD, and for other degenerative neurological diseases [46, 47]. Scaling up the transduction to spread throughout the human brain, as likely will be needed for AD patients, will be a challenge for this approach. Nonetheless, this study together with that by Fol et al. [20] provides a basis for greater investigations into methods for up-regulating sAPPα for treating AD and possibly other degenerative neurological disorders. In particular, treatment regimens beginning early in the disease process, together with biomarkers for identifying those with a high risk of developing AD, should be able to offer the greatest quality of life benefits for patients.

## Abbreviations

AAV: Adeno-associated virus
AD: Alzheimer’s disease
ANOVA: Analysis of variance
APPswe/PS1dE9: Amyloid precursor protein (Swedish mutation)/presenilin 1 (deleted exon 9)
Aβ: Amyloid-beta
DIV: Days in vitro
ELISA: Enzyme-linked immunosorbent assay
FEPSP: Field excitatory postsynaptic potential
GFP: Green fluorescent protein
I-O: Input-output
LTP: Long-term potentiation
LV: Lentivirus
NFκB: Nuclear factor kappa-light-chain-enhancer of activated B
PKG: Protein kinase G
PMSF: Phenylmethylsulfonyl Fluoride
PPF: Paired-pulse facilitation
PPI: Paired-pulse inhibition
SAPPα: Secreted amyloid precursor protein-alpha
TBS: Theta-burst stimulation
Tg: Transgenic
VSVg: Vesicular stomatitis virus
WT: Wild-type
